# Can a Targeted Pre‐Exercise Education Intervention Enhance the Exercise‐Induced Hypoalgesia (EIH) Response in Individuals With Knee Osteoarthritis (OA)?

**DOI:** 10.1111/1756-185x.70587

**Published:** 2026-03-10

**Authors:** David Toomey, Gwyn Lewis, Natalie Tuck, Ben Darlow, Usman Rashid, David Rice

**Affiliations:** ^1^ Auckland University of Technology Auckland New Zealand; ^2^ University of Otago‐Wellington Wellington New Zealand; ^3^ Waitematā Pain Services, Department of Anaesthesiology and Perioperative Medicine Te Whatu Ora Waitematā Auckland New Zealand

## Abstract

**Objective:**

Recent evidence suggests that education on the pain‐relieving effects of exercise may enhance exercise‐induced hypoalgesia (EIH) in healthy individuals. However, its impact in populations with osteoarthritis (OA), where EIH responses are more variable, remains unclear. This study examined whether positive pre‐exercise education enhances EIH in individuals with knee OA.

**Methods:**

A double‐blind, randomized controlled trial was conducted with 42 participants allocated to either a positive pre‐exercise education group (*n* = 21) or a control education group (*n* = 21). Each group received two individual education sessions 24–72 h apart. OA‐ and EIH‐related knowledge and beliefs were assessed pre‐ and post‐education. EIH was evaluated following a single submaximal isometric quadriceps contraction to failure by measuring changes in pressure pain thresholds (PPTs), resting pain, and pain during stepping. Group differences were analyzed using ANCOVA.

**Results:**

The positive pre‐exercise education group demonstrated greater improvements in EIH‐related knowledge and beliefs compared to the control group (*p* = 0.001, *d* = 0.50, ANCOVA between‐group analysis), while OA‐related knowledge and beliefs remained unchanged (*p* = 0.34, *d* = 0.15). However, ANCOVA results showed no significant between‐group differences in pre‐ to post‐exercise changes in PPTs, resting pain, or pain during stepping (all *p* > 0.11, *d* = 0.04–0.25).

**Conclusion:**

Despite enhancing beliefs about exercise‐induced pain relief, positive pre‐exercise education did not enhance EIH compared to control education. These findings highlight the need for alternative strategies to optimize exercise‐induced pain relief in OA.

## Significance and Innovations

- Positive pre‐exercise education enhances beliefs about EIH in knee OA but does not increase EIH magnitude compared to control education.
- Cognitive dissonance, arising from conflicting beliefs and lived experiences of pain during exercise, may limit the impact of brief educational interventions on EIH.
- Mechanisms underlying EIH extend beyond psychological factors, suggesting other interventions may be needed to optimize early pain relief with exercise in knee OA.

## 1 Background

Knee osteoarthritis (OA) is a prevalent and disabling condition, incurring substantial healthcare and economic costs [1]. Exercise is recommended as the first line of intervention by international evidence‐based guidelines [2], and is one of the most well‐documented interventions for alleviating pain and enhancing physical function in people with knee OA [3, 4]. Despite being universally recommended, exercise interventions continue to be underutilized by patients and clinicians [5, 6]. One reason for this is that the pain‐relieving effects of exercise are variable across individuals, both initially and over the course of an exercise programme [7, 8]. In particular, at the beginning of an exercise programme some individuals experience exercise‐induced flares in pain [9], which can negatively affect exercise adherence [10, 11].

Pain outcomes in knee OA are commonly assessed using a combination of subjective and experimental measures [12, 13]. Subjective pain measures capture the individual's self‐reported experience of pain intensity at rest or during movement (i.e., movement‐evoked pain) [13], while experimental measures such as pressure pain thresholds provide a quantitative measure of pain sensitivity [14]. These approaches capture complementary aspects of the pain experience and are frequently used to evaluate the effects of exercise on pain [15, 16].

A typical physiological response to exercise is exercise‐induced hypoalgesia (EIH), characterized by a short term reduction in pain and/or pain sensitivity that occurs following an acute bout of physical activity and may last for 5–30 min [12, 17]. In contrast, the EIH response is highly variable in individuals with knee OA, with studies demonstrating a normal EIH response [14, 15, 18, 19], an increase in pain sensitivity (hyperalgesic response) [13, 14, 20], or unchanged pain sensitivity [21]. The longer‐term response to exercise can also be variable in people with OA, with large population studies suggesting that approximately half of participants experience a moderate, clinically important (≥ 30%) improvement in self‐reported joint pain intensity, following an 8‐week program combining patient education and supervised neuromuscular exercise [22], while the other half do not. It has been suggested that the different pain responses to exercise programs seen in knee OA may be related to impaired EIH [14], with recent evidence demonstrating that reduced EIH, measured at baseline, predicts less improvement in pain and function over the course of 12 exercise sessions in people with knee OA [16].

Despite the potential importance of EIH in the management of pain, studies aiming to enhance EIH are sparse. In healthy, pain‐free controls, using brief targeted education (15 min) to positively modify expectations about the beneficial, analgesic, and safe effects of exercise was shown to increase EIH compared to a control education condition [23]. Furthermore, in another study of healthy pain‐free controls, negatively manipulating expectations (2–3 min) by suggesting a likely painful response with exercise decreased EIH responses compared to neutral or positive education interventions [24].

The effects of brief pre‐exercise education, designed to modify expectations of pain relief, have not been examined in an OA population. Such an intervention may be particularly relevant to knee OA, where negative beliefs, attitudes, and expectations about exercise, such as the belief that if exercise increases pain it will accelerate joint deterioration, are pervasive [25, 26].

Thus, the aim of this study was to investigate the impact of pre‐exercise education on EIH in people with knee OA. It was hypothesized that a larger EIH effect would be evident in individuals receiving targeted, positive education emphasizing the safety and pain‐relieving benefits of exercise in knee OA compared to a control education condition. Specifically, we hypothesized (1) a larger pre to post‐exercise increase in PPT, alongside greater pre to post‐exercise reductions in (2) resting pain and (3) movement‐evoked pain in the positive education group.

## 2 Materials and Methods

This parallel‐group, randomized controlled trial was conducted between September and December 2022. The 2010 Consolidated Standards of Reporting Trials (CONSORT) statement [27] and 2017 CONSORT of Non‐pharmacological Treatments Extension were used for reporting [28]. All procedures were approved by the Health and Disability Ethics Committee (21STH129) and Auckland University of Technology Ethics Committee (21/241), and written informed consent was obtained. The trial was registered with the Australian New Zealand Clinical Trials Registry (ANZCTR 12621000731897). All testing sessions were conducted at Auckland University of Technology's North Campus, Auckland, New Zealand.

### 2.1 Participants

Participants were recruited through advertisements and from existing research databases. Additionally, advertisements were left with local knee surgeons, rheumatologists, and physiotherapy clinics.

Inclusion criteria were: met The National Institute for Health and Care Excellence criteria for the diagnosis of knee OA (aged ≥ 45 years with activity‐related joint pain and either no morning stiffness or morning stiffness < 30 min), had ongoing knee pain for ≥ 3 months and an average knee pain intensity of ≥ 3/10 in the last week at the time of screening [29]. If participants had bilateral knee OA, the most painful knee was chosen as the index knee for testing. Exclusion criteria were: an inability to speak or write English, medical conditions preventing safe participation in physical activity, an inability to climb 2 flights of stairs, history of knee replacement, knee surgery in the past 6 months, a recent history of lower limb resistance training (≥ 2 times per week for a minimum of 6 weeks within the past 6 months), any other form of arthritis, a history of musculoskeletal pain or injury in the lower limb (other than OA) in the past 6 months, any neurological condition, a current diagnosis of a major psychiatric disorder, or any cognitive impairment.

### 2.2 Sample Size

A total of 42 participants were required (21 in each group) to achieve a probability of 80% that the study will detect a difference in EIH between interventions at a one‐sided 0.05 significance level, with an effect size of Cohen's *d* = 0.8. This calculation assumed a between‐group comparison of the primary outcome (change in PPTs from immediately before to after exercise) between two independent groups (independent‐samples *t*‐test framework). We utilized a one‐sided test as recommended when the alternative hypothesis is specific and directional (i.e., that EIH would improve with positive education compared to control education) [30]. A previous study investigating a similar pre‐exercise education intervention on the EIH response in a pain free population demonstrated a very large effect (*r* = 0.49 equating to Cohen's *d* of 1.12) [23]. We chose a more conservative effect size of Cohen's *d* = 0.8 to account for increased variability in the EIH response in people with OA [14].

### 2.3 Randomization

Participants were randomized in a 1:1 ratio using a computer‐generated randomization schedule (sealedenvelope.com) with permuted blocks of 2 and 6. To ensure allocation concealment, an independent researcher (not involved in recruitment or data collection) distributed and stored the randomization sequence in sealed, opaque envelopes. Participants, outcome assessors, and the statistician were blinded to treatment. Participants were informed the trial would “compare two different types of education and exercise interventions on knee osteoarthritis pain.” The intervention deliverer was not blinded by necessity.

### 2.4 Procedures

**FIGURE 1:**
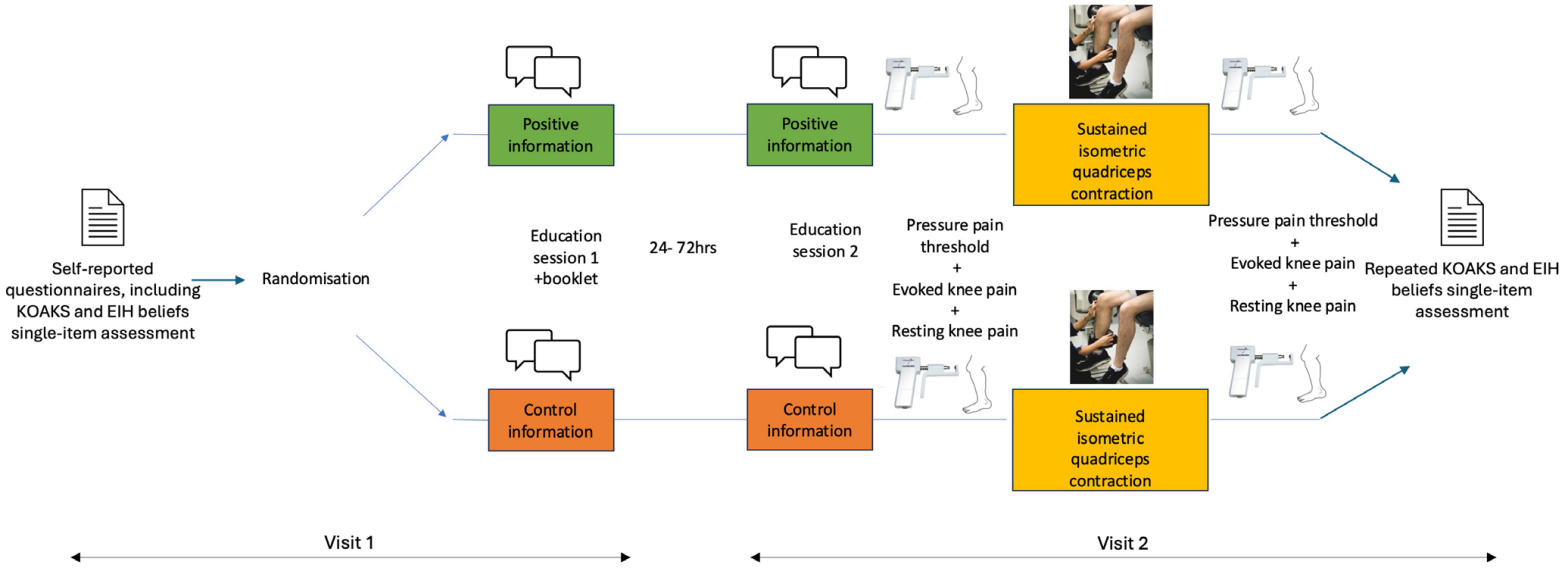
Experimental procedures. Participants were randomized to receive positive education about knee OA and EIH (intervention) or education about knee OA, exercise and pain (control). Each education session for both groups lasted approximately 30 min and participants were given a booklet to take home and read after the 1st visit. At the second visit, following the second education session, EIH was quantified by measuring the participant's pressure pain thresholds (PPTs), evoked knee pain, and resting knee pain before and after performing a sustained isometric quadriceps contraction at 25% of their maximum voluntary isometric contraction for 5 min, or until failure.

The experimental procedures are outlined in Figure 1. Each participant attended the laboratory during 2 clinical visits of ~2 h, 24–72 h apart (“visit 1” and “visit 2”). Participants were instructed to avoid vigorous exercise, alcohol, and caffeine for 24 h before testing and to refrain from nicotine use for at least 2 h prior to each testing session. During visit 1, participants provided demographic and clinical data for descriptive purposes, including age, sex, ethnicity, weight (kg), height (m), knee pain duration (years), and current medication use. Questionnaires regarding their pain, mental health, and physical function, as well as knowledge and beliefs related to pain and exercise, were completed. These included the Brief Pain Inventory (BPI) [31], Hospital Anxiety and Depression Scale (HADS) [32], Lower Limb Tasks Questionnaire [33], Pain Catastrophizing Scale (PCS) [34], Tampa Scale of Kinesiophobia (TSK) [35], Knee Osteoarthritis Knowledge Scale (KOAKS) [36] and a single item from Jones et al. [23] specifically related to EIH beliefs: “Pain can be reduced from just a single session of exercise” scored on a 7 point Likert scale from 0 = “strongly disagree” to 6 = “strongly agree.”

To standardize exercise intensity for visit 2 EIH testing, participants completed a quadriceps maximum voluntary isometric contraction (MVIC) assessment at the index knee using a Biodex System 3 dynamometer (Biodex Medical Systems, Shirley, NY, USA). Hip and knee flexion were fixed at 85° and 90° respectively. A standardized warm‐up included four 5‐s isometric contractions at 25%, 50%, 50%, and 75% of perceived maximum effort, followed by three 5‐s MVICs with 30‐s rests. Consistent verbal encouragement was provided, and MVIC was defined as the peak torque (Nm) achieved during any of the MVICs. Following MVIC testing, participants were familiarized with the upcoming visit 2 EIH testing procedure to obtain pain expectancy scores. They described their current resting knee pain (0–100 NPRS) and were told that in visit 2 they would maintain 25% of their MVIC torque for up to 5 min or until failure. They then performed a 10‐s trial at 25% MVIC and rated their expected knee pain (0–100 NPRS) if they held the contraction to failure. Pain expectancy was defined as the expected change in knee pain (expected pain—resting knee pain). All participants were then familiarized with the PPT testing procedures, which involved several practice trials on the dorsum of the hand to ensure understanding of the procedure and consistent reporting of pain onset. After baseline data collection, the envelope with their study number was opened for random allocation to either positive (intervention) or control pre‐exercise education. Participants then completed the first of two education sessions (visit 1).

During visit 2, participants first completed their second education session. Then, to quantify the EIH response, pressure pain thresholds (PPTs), resting and evoked knee pain were collected before and after a single bout of sustained, submaximal isometric quadriceps contraction (25% MVIC). Following the second education session but before exercise and EIH measures, both groups completed the KOAKS and a single‐item EIH beliefs questionnaire as a manipulation check for the interventions.

### 2.5 Interventions

Two 30‐min, one‐on‐one interactive education sessions (“Session 1” and “Session 2”), 24–72 h apart, were delivered by a postgraduate physiotherapist following standardized, rehearsed scripts (refined via pilot testing for Session 1 and adapted from Jones et al. [24] for Session 2; scripts and resources in Appendix 1). Sessions began with 10 min of rapport building. Participants received printed booklets covering Session 1 content to read between sessions. Engagement was encouraged through questioning to check understanding and relate content to personal experiences.

### 2.6 Positive Education

In the intervention group, Session 1 addressed common OA pain and management misconceptions with a biopsychosocial focus [14, 27]. Session 2 provided specific EIH education, describing pain reduction after exercise, effective exercise types, duration, and potential mechanisms, following a script from Jones et al. [24] with minor modifications (Appendix 1).

### 2.7 Control Education

In the control group, Session 1 used traditional, largely biomedical OA education from a reputable international website [37], with minor modifications to ensure neutrality regarding exercise and pain. The “Where can I find out more?” page was adapted for UK relevance. Session 2 covered pain ratings and differences in pain perception between athletes and nonathletes, following the control group script from Jones et al. [24].

### 2.8 Isometric Exercise

EIH was induced using a single submaximal isometric quadriceps contraction at 25% of MVIC on the Biodex dynamometer. Participants maintained this torque until failure (inability to sustain for ≥ 5 s) or a maximum of 5 min, with visual feedback provided. Rating of perceived exertion (RPE) on Borg's 6–20 scale was recorded every 30 s. Verbal encouragement was given until true failure or the 5‐min limit, and time to failure (seconds) was recorded. Participants then rated their maximum knee pain during the contraction (0–100 NPRS). Submaximal isometric exercise to failure was chosen due to its established effectiveness in producing EIH [13].

### 2.9 Primary Outcome Measures

The primary outcome was the change in PPTs from immediately before to after exercise. PPTs, a reliable and established measure in EIH research [17], were assessed at the index knee's medial joint line (MJL) (3 cm medial to the midpoint on the medial edge of patella) and the contralateral forearm (5 cm distal to the lateral epicondyle) in a randomized order pre‐ and post‐exercise, using the same order for both [14]. A handheld pressure algometer (SbMedic, Sweden) with a 1 cm rounded tip and a 30 kPa/s ramping rate was used. Participants pressed a button at the first onset of pain, and the pressure (kPa) was recorded. The average of three PPT measurements was taken per site. Both absolute and relative changes in PPTs at local (knee) and remote (forearm) sites were reported and analyzed as there is little consensus on which to report and both are frequently used [37, 38, 39]. Relative PPT change was expressed as a ratio of post‐ to pre‐exercise PPT; values > 1.0 indicated hypoalgesia, and < 1.0 indicated hyperalgesia [15].

### 2.10 Secondary Outcome Measures

Secondary outcomes included changes in resting and evoked knee pain intensity, assessed before and after exercise. Resting knee pain was measured using a 0–100 NPRS (0 = “no pain,” 100 = “worst imaginable”). Evoked pain was measured on the same scale before and after the Staircase‐Evoked Pain Procedure (StEPP): stepping up and down a 20 cm platform 24 times, starting with the index knee and alternating limbs [40]. Participants used their normal gait and were encouraged to complete the task without stopping. Time to complete the StEPP was recorded (seconds). Evoked pain was defined as the change in pain (0–100 NPRS) from before to immediately after the StEPP [40].

A fixed order of outcome measure testing was used to try to isolate the effects of exercise (EIH) on the outcome measures. Thus, before exercise, the order of assessment was evoked knee pain (StEPP test), 10 min rest, PPTs, then resting joint pain. A 10‐min rest period was given to allow any lingering effects of the StEPP test on pain sensitivity to dissipate before measurement of pre‐exercise PPTs. Immediately after exercise, the order was resting joint pain, PPT, and then evoked knee pain. Post‐exercise, resting joint pain was assessed immediately after exercise to capture exercise‐induced changes in resting pain levels, and because resting pain was quick to measure and would not influence the primary outcome, PPTs. PPTs were then immediately evaluated to determine the EIH response to exercise. Evoked knee pain (StEPP test) was performed last to ensure that any lasting effects of repeated joint loading did not influence the other post‐exercise outcomes.

### 2.11 Statistical Analysis

Normality of the data was assessed through visual inspection and using the Shapiro–Wilk statistic. As contraction duration (s), maximum knee pain during contraction, and peak RPE were non‐normally distributed, these outcomes were compared across groups using Mann–Whitney *U* tests to check that the exercise dose was similar. For each group, Wilcoxon signed rank tests were undertaken to ensure that the time taken to complete the evoked pain test (StEPP) was similar before compared to after exercise.

For primary and secondary outcome measures, a two‐step analysis of covariance (ANCOVA) approach was used. In the first step, a full model regressed post‐exercise (or change) scores on pre‐exercise scores and all prespecified covariates (age, anxiety subscale of the HADS, and expected change in pain) [41]. These covariates were chosen based on the findings of a large prospective cohort study exploring predictors of EIH in a knee OA population [42]. To account for baseline differences between groups, the baseline single item belief scores were added as an additional covariate. Multicollinearity was evaluated for all the covariates with the variance inflation factor (VIF) [43]. Any variable with a VIF score greater than 10 was excluded. Mean differences across groups (the treatment effect) and mean score or mean change scores within each group (pre‐ to post‐intervention effects), along with 95% confidence intervals (CIs) are reported. To complement these adjusted analyses, unadjusted one‐way ANOVAs on pre‐ to post‐exercise change scores were also performed and presented in Appendix S1. These confirm that the pattern of results was unchanged when covariates were not included.

Between group effect sizes were calculated by dividing the mean difference between groups by the pooled standard deviation and interpreted according to Cohen's criteria of 0.2 = small, 0.5 = medium, and 0.8 = large [44]. To examine the potential relationship between beliefs about EIH and the magnitude of EIH, post hoc exploratory correlation analyses were undertaken. These analyses examined the relationship between the primary outcome measure (pre‐to‐post exercise absolute change in PPT) and: (1) the pre‐to‐post intervention change in EIH beliefs and (2) the absolute post intervention EIH beliefs score using Spearman's rank correlation coefficient.

The data were analyzed using the R environment for statistical computing [45, 46] and SPSS version 25 (IBM Corp, Armonk, NY). Statistical significance was defined as *p* ≤ 0.05 for all analyses.

## 3 Results

### 3.1 Participant Characteristics

**FIGURE 2:**
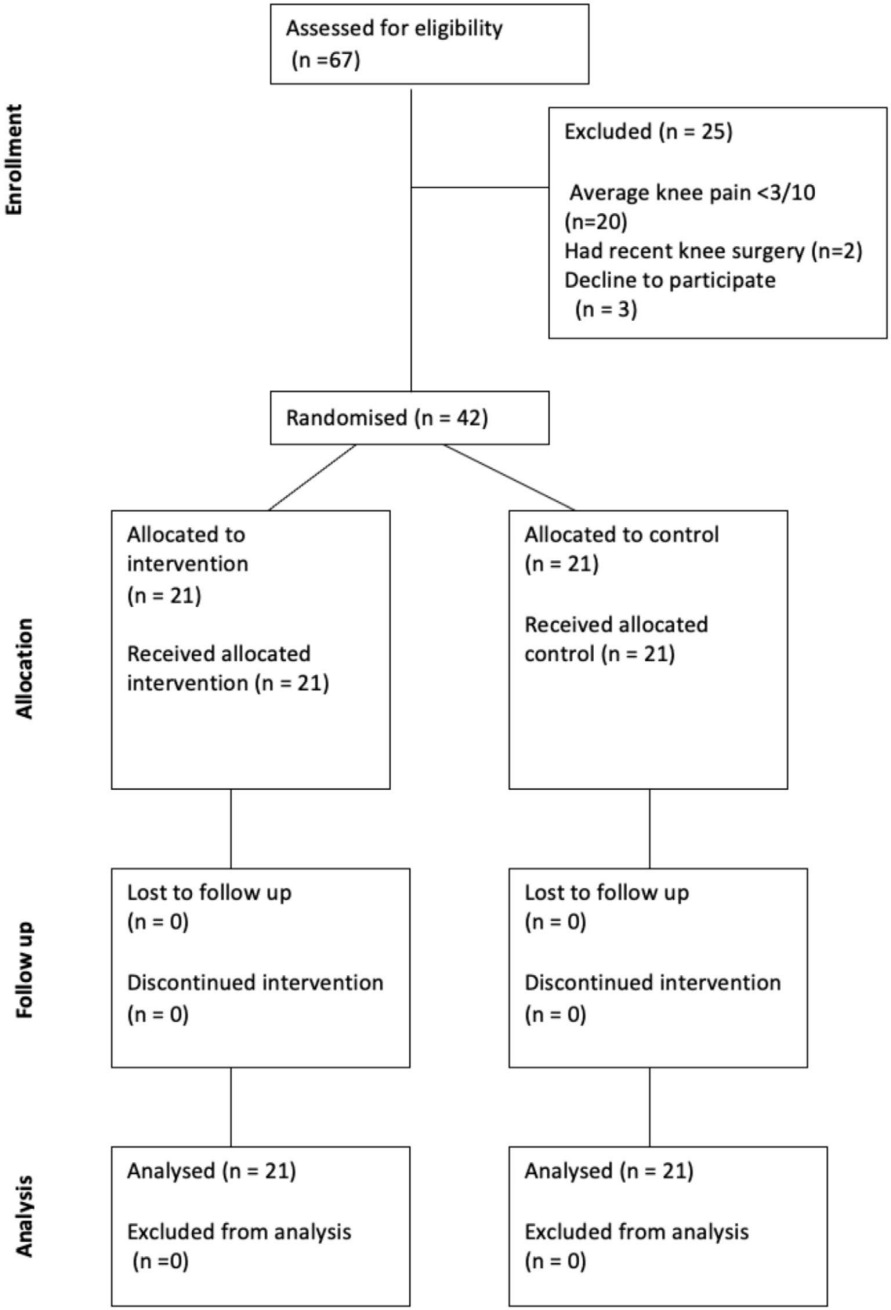
CONSORT flow diagram of the progress through the phases of the randomized trial for each group.

A total of 67 participants were screened for eligibility, of which 25 (39%) were excluded for being ineligible, while 42 participants (65%) (20 females, 22 males) with an average age of 66 ± 7.5 years were recruited (Table 1, Figure 2). Baseline characteristics of the two groups were similar (Table 1). All participants completed the PPT testing, evoked pain testing (StEPP test), and sustained isometric contraction, with no adverse events reported.

**TABLE 1:** Baseline participant characteristics.

	Intervention group	Control group
Sample size (*n*)	21	21
Age	65 (8.6)	67 (6.2)
Female sex (%)	13 (62%)	9 (43%)
Ethnicity: frequency (%)		
New Zealand European	16 (76%)	16 (76%)
New Zealand Māori	1 (5%)	0 (0%)
Samoan	0 (0%)	1 (5%)
Other European	3 (14%)	3 (14%)
Other ethnicity	1 (5%)	1 (5%)
Height (cm)	170 (7)	174 (10)
BMI (kg/m^2^)	28.4 (6.0)	30.1 (5.8)
Duration of knee pain (months)	60 (18–138)	72 (36–120)
LLTQ (0–100)	18 (14–27)	21 (14.5–25)
HADS‐depression (0–21)	3 (2–5.5)	4 (1–5.5)
HADS‐anxiety (0–21)	5 (2–8.5)	4 (2.5–7)
TSK (11–44)	30 (21–31.5)	29 (25–32.5)
PCS (0–52)	8 (4–10)	5 (4–14)
BPI‐Ave (0–10)	3 (1.5–4)	3 (2–4.5)
BPI‐worst (0–10)	5 (3–7)	5 (3–7.5)
BPI‐least (0–10)	0 (0–1.5)	0 (0–1)
BPI‐interference (0–10)	2.6 (1.6–5.1)	2.7 (1.9–5.4)
Peak torque (Nm)	142 (108–175)	160 (120.5–173.5)
Single item EIH beliefs (0–7)	2 (1–4)	3 (1–5)
KOAKS (11–55)	33 (33–34)	36 (34–39)
EIH testing order (knee first (%))	12 (57%)	9 (43%)
Taking any pain medication (%)	3 (14%)	2 (10%)
Types of regular pain medication: frequency (%)		
Paracetamol	1 (5%)	0 (0%)
Anti‐inflammatories	1 (5%)	2 (10%)
Opioids	0 (0%)	0 (0%)
Anticonvulsants	1 (5%)	0 (0%)
Antidepressants	0 (0%)	0 (0%)

*Note:* Data are presented as mean (SD) or median (IQR) unless otherwise stated.

Abbreviations: BMI, body mass index; BPI, Brief Pain Inventory; HADS, Hospital Anxiety and Depression Scale; IQR, Interquartile range; KOAKS, Knee Osteoarthritis Knowledge Scale; LLTQ, Lower Limb Task Questionnaire; m, meters; min, minutes; PCS, Pain Catastrophizing Scale; s, seconds; SD, Standard deviation; TSK, Tampa Scale for Kinesiophobia.

### 3.2 Similarity of the Exercise Interventions and StEPP Test Performance

There were no significant between‐group differences in time to failure (*p* = 0.51), maximum knee pain during exercise (*p* = 0.50), or peak RPE (*p* = 0.81) during the sustained isometric contraction (Table 2). The time taken to complete the StEPP test did not differ before and after isometric exercise in the control group (*p* = 0.20). However, in the intervention group, StEPP test performance was marginally faster post‐exercise compared to pre‐exercise (*p* = 0.05).

**TABLE 2:** Expected change in pain, target torque, peak rating of perceived exertion (RPE), time to failure, maximum knee pain during contraction on the Numerical Pain Rating Scale (NPRS), and StEPP time to completion across the intervention and control groups.

Measure	Intervention group	Control group	*p*
Expected change in pain NPRS (0–100)	34.1 (27.2)	28.5 (26.5)	0.50
Target torque (Nm)	36.0 (12.5)	38.4 (10.9)	0.48
Peak RPE (6–20)	20 (19–20)	20 (19–20)	0.81
Time to failure (s)	300 (241.75–300)	300 (251.25–300)	0.51
Maximum knee pain during contraction NPRS (0–100)	27.5 (0.5–47.50)	27.5 (0.0–57.50)	0.50
StEPP time to completion (pre‐intervention) (s)	65 (59–75.5)	75.5 (66.75–86.75)	
StEPP time to completion (post‐intervention) (s)	63.5 (55.5–74)	70.5 (65.25–88)	

*Note:* Values are displayed as mean (SD) or median (IQR) unless otherwise stated.

Abbreviations: IQR, interquartile range; NPRS, Numerical Pain Rating Scale; RPE, Rating of perceived exertion; SD, standard deviation.

### 3.3 Manipulation Checks

The pre‐ to post‐intervention change in EIH‐related beliefs was greater in the positive education group compared to the control education group, with a medium effect (mean difference = 1.3, 95% CI [0.5, 2.1], *p* = 0.001, *d* = 0.50; Table 3). In contrast, while the change in OA‐related knowledge and beliefs assessed by the KOAKS questionnaire favored the positive education group with a very small effect, this did not reach statistical significance (mean difference = 1, 95% CI [−1, 3], *p* = 0.34, *d* = 0.15). Neither group significantly increased their KOAKS score from pre‐ to post‐intervention (both *p* > 0.22).

**TABLE 3:** Pre‐ to post‐education intervention change scores in single item exercise‐induced hypoalgesia (EIH) beliefs and Knee Osteoarthritis Knowledge Scale (KOAKS).

Measure	Intervention group	Control group	Between group difference (95% CI)	*p*
EIH beliefs (0–7)	2.1 (0.3)*	0.8 (0.3)*	1.3 (0.5, 2.1)	0.001
KOAKS (11–55)	0.9 (0.7)	−0.1 (0.7)	1 (−1, 3)	0.34

*Note:* Values are displayed as estimated marginal means (standard error) unless otherwise stated.

Abbreviations: CI, Confidence interval; EIH, Exercise Induced Hypoalgesia; KOAKS, Knee Osteoarthritis Knowledge Scale; SE, Standard error.

* Represents significant within group change from pre‐to post‐intervention *p* < 0.05.

### 3.4 Primary Outcomes

Analysis of the absolute change in PPT revealed no significant differences between the positive and control education groups at the knee (mean difference 10 kPa [95% CI −40 to 60 kPa]; *p* = 0.57, *d* = 0.08) or the forearm (mean difference −10 kPa [95% CI −50 to 30 kPa]; *p* = 0.56, *d* = −0.08). Similarly, there was no between‐group difference in the relative change in PPT at either the knee (mean difference 0 [95% CI −0.2 to 0.19]; *p* = 0.97, *d* = 0) or the forearm (mean difference 0 [95% CI −0.3 to 0.2]; *p* = 0.90, *d* = 0). Unadjusted one‐way ANOVA results were consistent with the adjusted analyses, showing no significant between‐group differences in pre‐to‐post changes across all pain outcomes (Appendix S1).

**FIGURE 3:**
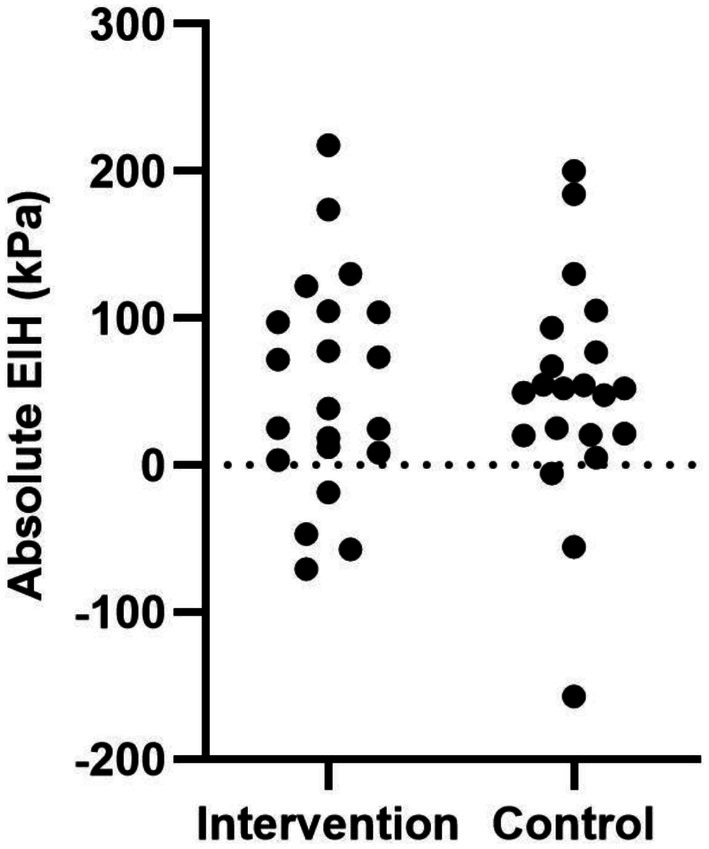
Distribution of the absolute change in knee pressure pain thresholds from pre‐ to post‐isometric exercise for individual participants at the knee (local site) in the intervention and control groups. Positive values indicate hypoalgesia and negative values indicate hyperalgesia, with the dotted line representing no change from baseline.

On average, both groups demonstrated a significant increase in knee PPT following isometric exercise, reflecting a local EIH response (knee). Remote EIH (forearm) was smaller, and only statistically significant when expressed as a relative change (Table 4). Despite a significant local EIH response at the knee, the variability in the EIH response was large in both the positive and control education groups (Figure 3).

**TABLE 4:** Primary and secondary outcome measures across the intervention and control groups.

Measure	Intervention group	Control group	Between group difference [95% CI]	*p*
Knee PPT				
Pre‐exercise (kPa)	317 (231)	241 (177)		
Post‐exercise (kPa)	370 (266)*	291 (167)*		
Absolute EIH (kPa)	60 (20)	40 (20)	10 [−40, 60]	0.57
Relative EIH (ratio)	1.24 (0.07)*	1.24 (0.07)*	0 [−0.2, 0.19]	0.97
Forearm PPT				
Pre‐exercise (kPa)	281 (103)	315 (140)		
Post‐exercise (kPa)	309 (205)	319 (152)		
Absolute EIH (kPa)	10 (10)	20 (10)	−10 [−50, 30]	0.56
Relative EIH (ratio)	1.07 (0.08)	1.09 (0.08)	0 [−0.3, 0.2]	0.90
Resting pain (0–100 NPRS)				
Pre‐exercise	11.48 (13.32)	8.05 (11.07)		
Post‐exercise	9.95 (14.71)	12.95 (18.62)		
EIH (resting pain change)	−2 (3)	6 (3)	−8 [−18, 2]	0.11
Evoked pain (0–100 NRPS)				
Pre‐exercise	15.14 (17.69)	12.19 (11.87)		
Post‐exercise	11.67 (21.05)	9.00 (11.32)		
EIH (evoked pain change)	−3 (3)	−4 (3)	1 [−7, 8]	0.90

*Note:* Pre‐ and post‐exercise values are displayed as mean (standard deviation) or median (interquartile range), while change scores are estimated marginal means (standard error) or median (interquartile range).

Abbreviations: CI, Confidence interval; kPa, Kilopascals; NPRS, Numeric Pain Rating Scale; PPT, Pressure Pain Threshold.

* Represents significant within session change from pre‐to post‐intervention *p* < 0.05.

### 3.5 Secondary Outcomes

There was no significant between‐group difference in the pre‐ to post‐exercise change in resting pain (mean difference −8 [95% CI −18 to 2]; *p* = 0.11, *d* = −0.25) or evoked pain (mean difference 1 [95% CI −7 to 8]; *p* = 0.90, *d* = 0.04) (Table 4).

### 3.6 Relationship Between EIH Beliefs and Primary Outcomes

For the entire sample, there were no significant relationships between absolute or relative EIH (∆ in PPTs) and either the pre‐to‐post‐intervention change, nor the absolute post‐intervention score of the single item EIH beliefs question (rho −0.18 to 0.23, all *p* > 0.26).

## 4 Discussion

To our knowledge, this is the first study to investigate the effect of pre‐exercise education on EIH in individuals with knee OA. Previous reviews have shown that while EIH is a consistent and robust phenomenon in pain‐free populations, its presence and magnitude in chronic pain conditions, including OA, are highly variable [12, 17, 47]. Some studies in people with OA report preserved hypoalgesia following exercise [14, 15, 18, 19] whereas others show no change [21], or even hyperalgesic responses [13, 14, 20], mirroring the variability in pain relief observed with exercise in this population.

The current study shows that, compared to a control condition, positive pre‐exercise education leads to an increase in the belief that a single exercise session can reduce pain but does not affect overall knee OA knowledge as measured by the KOAKS, nor the magnitude of the subsequent EIH response. This includes EIH assessed as the change in PPT (primary outcome) as well as EIH assessed as the change in resting knee pain and evoked knee pain (secondary outcomes). Furthermore, there was no significant association between the change in PPTs and EIH‐related beliefs.

Despite utilizing a near‐identical script for the education interventions, our results differ from the findings of Jones et al. [23], who observed that in healthy, pain‐free control participants, positive pre‐exercise education enhanced EIH when compared to a control pre‐exercise education, with a large effect size. In addition, a significant positive correlation was observed in their study between the magnitude of the post‐intervention EIH response and the degree to which participants agreed with the single item question “pain can be reduced from just a single session of exercise” [23].

Our findings are more in line with Vaegter et al. [24], who demonstrated that brief (2–3 min) positive pre‐exercise education did not increase EIH in healthy pain‐free controls. Conversely, they found that negative pre‐exercise education diminished EIH. This observation is consistent with the concept that negative experiences tend to exert a more profound influence on psychological states than positive ones [48], which may in turn influence endogenous pain modulation pathways. It could be that the educational interventions of our study were not sufficiently dissimilar to elicit differential outcomes on EIH. Our decision to avoid a negative intervention, despite its potential to enhance the difference between groups, was based on the ethical consideration to avoid nocebic effects in a clinical population suffering from chronic pain. Furthermore, our focus was on whether providing positive education offers benefits compared to standard biomedical education. This arguably has more clinical relevance than simply establishing its superiority over deliberately nocebic approaches, as it aims to direct treatment strategies toward interventions that do more than avoid harm but rather actively contribute to patient care.

It is well established that people with knee OA commonly have misperceptions about their condition and frequently hold negative attitudes and beliefs about exercise [49]. These may be resistant to change, particularly in the short term. Despite our positive education intervention increasing confidence that “pain can be reduced from just a single session of exercise,” this may not have aligned with the personal experiences of participants, especially if they have previously found exercise to be painful or challenging. This mismatch, a form of cognitive dissonance, reflects the complex decision‐making process knee OA patients face, as described by Darlow et al. [26], balancing knowledge of the therapeutic role of exercise with the fear of increasing pain or causing further joint damage.

Alternatively, it may be that impaired EIH is only weakly related to expectations in people with knee OA. The mechanisms of EIH remain incompletely understood and are thought to be mediated by a range of different physiological processes [12, 50]. It has been suggested that impaired EIH may reflect maladaptive changes in the function of endogenous pain inhibitory pathways and/or immune system dysfunction [12]. Perhaps, in the presence of such maladaptive changes, cognitive factors such as expectations are less important in determining the overall magnitude of the EIH response. This is supported by a recent systematic review which suggests that cognitive and emotional factors appear to have a stronger association with EIH in pain‐free individuals than in those with chronic pain conditions [51].

Strengths of this study include its robust double‐blind RCT design and the inclusion of secondary outcome measures of EIH that may have more direct clinical relevance than PPT alone. However, there are also some potential limitations to consider. Notably, an isometric exercise protocol was employed to induce EIH, while Jones et al. [23] used an aerobic exercise protocol. The selection of an isometric protocol is arguably more clinically relevant as local resistance training is routinely prescribed in the management of OA. Previous studies have shown that aerobic and isometric exercise elicit EIH of a similar magnitude, both in healthy controls [17] and knee OA specifically [14]. Thus, it seems unlikely that the type of exercise used to elicit EIH would have affected our results, although this remains a possibility. Importantly, our population of people with OA appeared to have mild to moderate symptoms; we did not collect data on participant comorbidities, and we only examined the immediate effects of an education intervention on EIH after a single bout of exercise. Therefore, our findings may not translate to all people with knee OA or to the longer‐term effects with repeated bouts of exercise, which should be examined in future studies. For example, over the course of several sessions, the combination of pain neuroscience education and exercise has been shown to lead to greater improvements in pain than exercise alone [52]. Finally, there was a statistically significant difference in the time taken to perform the StEPP test from pre to post exercise in the intervention group. However, the mean difference was marginal (~1.5 s) and similar to the pre to post exercise difference observed in the control group. As such, it is unlikely to have had an important effect on the findings.

## 5 Conclusions

Despite modifying EIH related beliefs, two 30‐min sessions of positive pre‐exercise education did not enhance the EIH response compared to control pre‐exercise education. Different types of interventions, more intensive pre‐exercise education interventions, or similar interventions in participants with more severe symptoms should be explored to see if they enhance EIH in people with knee OA. This may minimize exercise‐induced flares in pain and increase both efficacy and engagement with exercise‐based rehabilitation in the longer‐term.

## Author Contributions

This study was conceived and designed by D.T., G.L., B.D., U.R., N.T., and D.R. Data collection was performed by D.T. Data analysis and interpretation were conducted by D.T. and D.R., with input from U.R., D.T. prepared the first draft of the manuscript. All authors have read and agreed to the published version of the manuscript.

## Funding

The authors have nothing to report.

## Conflicts of Interest

The authors declare no conflicts of interest.

## Supporting information


**Appendix S1:** Active Control: Script for the education of participants with knee osteoarthritis, utilising the “Versus Arthritis: Osteoarthritis of the knee” handbook, excluding explicit information of exercise induced hypoalgesia.

## Data Availability

The data that support the findings of this study are available from the corresponding author upon reasonable request.
