# Supplementary material for: Can a Targeted Pre‐Exercise Education Intervention Enhance the Exercise‐Induced Hypoalgesia (EIH) Response in Individuals With Knee Osteoarthritis (OA)?
Source: Int J Rheum Dis. 2026 Mar 10;29(3):e70587. doi: 10.1111/1756-185x.70587 (PMC12972853; doi:10.1111/1756-185x.70587)

Appendix 1. *Supplementary Material*

Active Control: Script for the education of participants with knee osteoarthritis, utilising the "Versus Arthritis: Osteoarthritis of the knee" handbook, excluding explicit information of exercise induced hypoalgesia.

Participant has been deemed eligible to participate in the study and arrives at the clinic and informed consent and introduction formalities are completed.

<u>Session 1 (30 min)</u>

At the beginning of session 1, the experimenter will introduce himself to the participant. Following this approximately 5 minutes will be spent on rapport building in order to help the particiapnt feel comfortable, build trust, increase confidence and form a therapeutic relationship.

Experimenter: "*When did you start having trouble with your knee?"*

Participant responds:

Experimenter: Acknowledges any key points and then.

"Would you be able to tell me in your own words, what is your understanding of knee OA?"

Participant responds:

Experimenter: Answers any questions raised by the participant and reflects on any relevant points using the handbook and then:

"How does that sound to you?"

Participant responds:

Experimenter: Acknowledges any key points and then.

"OA can affect people in a range of ways. What do you notice specifically about how it affects you?

Participant responds:

Experimenter: Answers any questions raised by the participant and reflects on any relevant points using the handbook and then:

"How does that sound to you?"

Participant responds:

Experimenter: Acknowledges any key points and then.

"What helps your knee? .... How does this help ?"

Participant responds:

Experimenter: Answers any questions raised by the participant and reflects on any relevant points using the handbook and then:

"How does that sound to you?"

Participant responds:

Experimenter: Acknowledges any key points and then.

"What do you do when your knee feels sore?" Why is that?"

Participant responds:

Experimenter: Answers any questions raised by the participant and reflects on any relevant points using the handbook and then:

"How does that sound to you?"

Participant responds:

Experimenter: Acknowledges any key points and then.

"What do you think you should be doing that you don't? .... Why don't you?"

Participant responds:

Experimenter: Answers any questions raised by the participant and reflects on any relevant points using the handbook and then:

"How does that sound to you?"

Participant responds:

Experimenter: Acknowledges any key points and then.

"I would now like to chat about some support resources that are available."

Experimenter: Talks through support options section as per Versus handbook then:

Experimenter: *"If you were to summarise what we chatted about today with your family/partner, what would you tell them?"*

Participant responds:

Experimenter: Answers any questions raised by the participant and reflects on any relevant points using the handbook and then:

"Alright, well I hope that information was useful for you and that you have learned something new about knee osteoarthritis. Now I would just like to quickly explain what is going to happen during your next session with me in a few days where we will be discussing exercise and performing some tests."

Following description of session 2 procedures:
Experimenter: *"Thank you again for your time and for agreeing to participate in this study. I look forward to seeing you again for session 2."*
Session 2 (30 min) script

At the beginning of session 2, the experimenter will again spend approximately 5 minutes on rapport building in order to help the particiapnt feel comfortable, build trust, increase confidence and form a therapeutic relationship.

Experimenter: *"Thanks again for coming along for your second session. To start things off, is there anything from the booklet or any questions you have having had a look through your booklet over the last few days?"*

Participant responds:

Experimenter: Summarise key points about modality and frequency and then ask:

Experimenter: *"Today we are going to focus more on exercise. Can you tell me what type of exercise or activity?, if any, you regularly participate in and how often?"*

Participant responds:

Experimenter: Summarise key points about modality and frequency and then ask:

"Have you ever engaged in exercise or activity that is intense enough to cause pain during or

immediately following the exercise?"

Participant responds:

Experimenter: Summarise relevant information and add

"Muscle pain during and immediately following strenuous exercise or activity is called acute muscle soreness and generally disappears shortly after finishing exercise. The pain is the result of energy metabolism, often referred to as 'metabolites' and one of these you might have heard of is lactic acid. The increase in these metabolites in our muscles sends stress signals to the brain, which eventually contribute to feeling exhausted from exercise. The feeling of exhaustion prompts us to rest and to let our muscles recover. So the pain during exercise has a role to protect us from exercising for too long or too hard. Are you familiar with this type of muscle pain during exercise?"

Participant responds:

Experimenter: Summarise relevant information and, with the aid of Figure 1, adds:

"Another type of muscle pain that occurs after strenuous or unaccustomed exercise or physical activity is termed delayed onset muscle soreness, or DOMS. The pain associated with DOMS often gets worse in the 24-72 hours following exercise before slowly reducing. The pain is thought to be caused by micro trauma to the muscle fibres. You're particularly likely to get DOMS if you've been walking or running up and down hills. Have you ever experienced DOMS?"

Participant responds:

Experimenter: Responds to the participant's answer and adds:

"While DOMS can be quite painful, the muscles adapt quickly. If you get DOMS once from some exercise you probably won't get DOMS the second time you do that exercise – or at least not nearly so bad. It just indicates that our muscles are repairing after some unaccustomed exercise and are getting stronger. While most people know about how good regular exercise is for things like managing weight, improving lung and heart function, and even our mood, what we are starting to learn is that even a single bout of exercise can provide physical and psychological benefits. Have you come across much information about the benefits of just a single bout of exercise?

Participant responds:

Experimenter: Summarises key points raised by the participant and affirms these with the participant:

Participant responds:

Experimenter: Reflects on any relevant points the participant has raised and then:

"Yes, some research has shown that even short bouts of exercise, as little as 5 minutes, can improve fitness, health and wellbeing. However, some people who decide to commence more strenuous exercise experience acute muscle soreness or DOMS and this often discourages them from further participation in exercise. This study that you have volunteered to take part in is about pain during a bout of normal

intensity exercise for 5 mins and requires me to explain a bit more to you about pain during this type of exercise. I'll start by asking you a question. When you exercise, how do you determine if it is safe to continue if your pain increases or do you see the increase in pain as an indication to stop?"

Participant responds:

Experimenter: Summarise any relevant information provided and then:

"It's normal to feel an increase in discomfort during exercise and this is not an indication that you are causing further damage to the muscle or that you are

hurting yourself. It is safe to continue to exercise when the increases in pain you experience are tolerable and feel manageable. This discomfort should level out during exercise and reduce shortly after you finish. If you feel the muscles are getting tired or hurting too much during the exercise, then you should just drop the intensity slightly back to an easier level. You can apply this to the exercise bout you are about to undertake for this study. Before we continue, do you have any questions about pain and discomfort during exercise?"

Participant responds:

Experimenter: Answers any of the questions raised by the participant and then:

"I would now like to talk about how levels of pain and exertion are typically

measured during exercise. Do you know anything about this?"

Participant responds:

Experimenter: Reflects on any relevant points the participant has raised and then:

"Because pain and exertion are both things that only you can feel, we ask you to tell us about these using rating scales .For example, I might ask you to rate your pain during exercise on a 0 to 100 scale whereby 0 is no pain and 100 is the worst possible pain. I could use a similar scale to ask you about your level of exertion during exercise, and this information would be useful for me to know how hard you are finding the exercise. Have you used these types of scales before?"

Participant responds:

"Another useful aspect of these types of scales is that they can be used to assess different aspects of pain such as pain intensity and pain unpleasantness. Pain intensity describes how strong the pain is whereas pain unpleasantness describes how bothersome it is and just because something is intense, that doesn't necessarily mean that it is bothersome and vice versa. For example, you might find the pain from a hard massage to be quite intense but not necessarily unpleasant. There is some interesting research in athletes using these different types of pain ratings showing that sportsmen and women typically have lower ratings of pain

unpleasantness and higher pain tolerances compared to non-athletes. Basically, athletes find things to be just as painful but are willing to tolerate them for longer. This is probably a big part of why endurance athletes like marathon runners and cyclists are able to exercise at high intensities for so long. Now I don't wish to create an impression that with exercise we can all become stoical like elite athletes and learn to ignore pain. The point is simply that we recognise the different aspects of the pain experience and how these interact with exercise. Do you have any questions about this?"

Participant responds:

Experimenter: summarises key points raised by the participant and then:

"Okay, well it's good that you are now a little more familiar with some of the causes of pain during exercise as well as how pain and exertion are measured during exercise as you are going to be asked to rate these sensations later on when you are exercising. Is it okay if I summarise the key points we talked about before we commence exercise?"

Key points on a card:

- *It is normal to experience some muscle pain and discomfort during and for a short time following exercise and pain during exercise doesn't mean that you are causing lasting damage or injury.*

- *It is also normal to experience longer lasting muscle pain, or DOMS, after intense or unaccustomed exercise, but the muscles gradually adapt to this and the pain subsides*

- *During exercise at an intensity that causes some discomfort, tolerable increases in pain are normal and safe. We can monitor these increases in pain and discomfort during exercising using self-report scales.*

"I just have a few final questions before we go on with the rest of the experiment."

[Experimenter presents the 5 questions below and asks the participant to indicate their level of agreement with each question. The experimenter then circles their response]

Experimenter: *"Thank you. Do you have any questions before we commence?"*
Participant responds:

Experimenter: *"Alright, well I hope that information was useful for you and that you have learned something about pain during exercise. Now I would just like to quickly explain what is going to happen for the rest of the experiment, after which I will hand over to one of my colleagues who take you through the exercise bout and pain assessments."*

Following description of experimental procedures:

Experimenter: *"Thank you again for your time and for agreeing to participate in this study. I will leave you with my colleague and see you again when you're done."*

Delayed Onset Muscle Soreness (DOMS)

What is it?

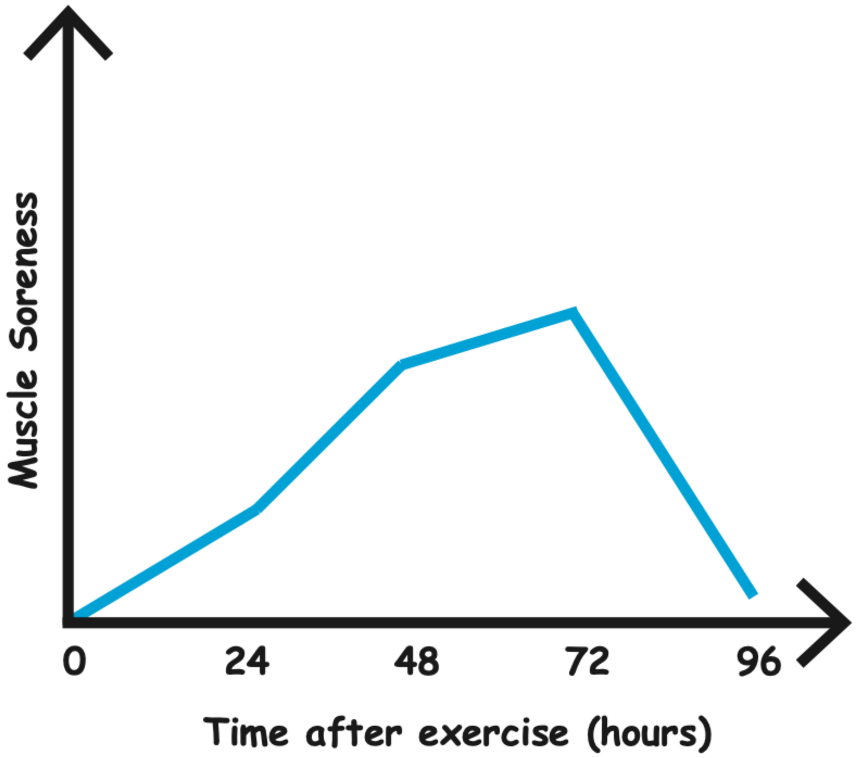

Why does it occur?

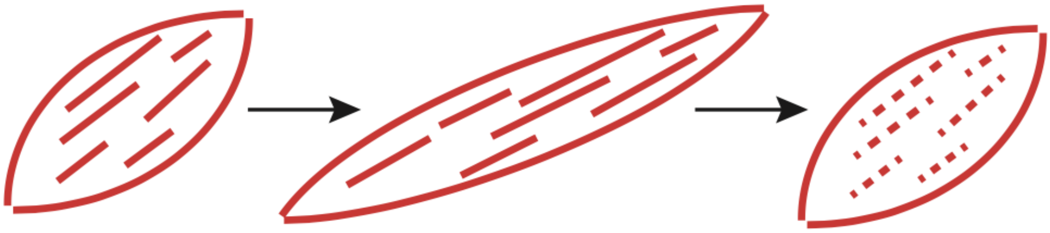

Figure 1

Intervention: Script for the education of participants with knee osteoarthritis, utilising the "Information for people with Knee Arthritis" handbook, including explicit information of exercise induced hypoalgesia.

Participant has been deemed eligible to participate in the study and arrives at the clinic and informed consent and introduction formalities are completed.

Session 1 (30 min)

At the beginning of session 1, the experimenter will introduce himself to the participant. Following this approximately 5 minutes will be spent on rapport building in order to help the particiapnt feel comfortable, build trust, increase confidence and form a therapeutic relationship.

Experimenter: ***"****When did you start having trouble with your knee?"*

Participant responds:

Experimenter: Acknowledges any key points and then.

"Would you be able to tell me in your own words, what is your understanding of knee OA?"

Participant responds:

Experimenter: Answers any questions raised by the participant and reflects on any relevant points using the handbook and then:

"How does that sound to you?"

Participant responds:

Experimenter: Acknowledges any key points and then.

"OA can affect people in a range of ways. What do you notice specifically about how it affects you?

Participant responds:

Experimenter: Answers any questions raised by the participant and reflects on any relevant points using the handbook and then:

"How does that sound to you?"

Participant responds:

Experimenter: Acknowledges any key points and then.

"What helps your knee? .... How does this help ?"

Participant responds:

Experimenter: Answers any questions raised by the participant and reflects on any relevant points using the handbook and then:

"How does that sound to you?"

Participant responds:

Experimenter: Acknowledges any key points and then.

"What do you do when your knee feels sore?" Why is that?"

Participant responds:

Experimenter: Answers any questions raised by the participant and reflects on any relevant points using the handbook and then:

"How does that sound to you?"

Participant responds:

Experimenter: Acknowledges any key points and then.

"What do you think you should be doing that you don't? .... Why don't you?"

Participant responds:

Experimenter: Answers any questions raised by the participant and reflects on any relevant points using the handbook and then:

“How does that sound to you?”

Participant responds:

Experimenter: Acknowledges any key points and then.

“I would now to chat about some support resources that are available.”

Experimenter: Talks through support options section as per handbook then:

Experimenter: *"If you were to summarise what we chatted about today with your family/partner, what would you tell them?"*

Participant responds:

Experimenter: Answers any questions raised by the participant and reflects on any relevant points using the handbook and then:

“Alright, well I hope that information was useful for you and that you have learned something new about knee osteoarthritis. Now I would just like to quickly explain what is going to happen during your next session with me in a few days where we will be discussing exercise and performing some tests.”

Following description of session 2 procedures:
Experimenter: *“Thank you again for your time and for agreeing to participate in this study. I look forward to seeing you again for session 2.”*

Session 2 (30 min) script

Experimenter: *“Thank you for coming along for your second session. To start things off, is there anything from the booklet or any questions you have having had a look through your booklet over the last few days?”*

Participant responds:

Experimenter: Summarise key points about modality and frequency and then ask:

Experimenter: *“Today we are going to focus more on exercise. Can you tell me what type of exercise, if any, you regularly participate in and how often?”*

Participant responds:

Experimenter: Summarise key points about modality and frequency and then ask:

"Have you ever engaged in exercise that is intense enough to cause pain during or immediately following the exercise?"

Participant responds:

Experimenter: Summarise relevant information and add

"Muscle pain during and immediately following strenuous exercise is called acute muscle soreness and generally disappears shortly after finishing exercise. The pain is the result of end products of energy metabolism, often referred to as 'metabolites' and one of these you might have heard of is lactic acid. The increase in these metabolites in our muscles sends stress signals to the brain, which eventually contribute to feeling exhausted from exercise. The feeling of exhaustion prompts us to rest and to let our muscles recover. So the pain during exercise has a role to protect us from exercising for too long or too hard. Are you familiar with this type of muscle pain during exercise?"

Participant responds:

Experimenter: Summarise relevant information and add

"Another type of muscle pain that occurs after strenuous or unaccustomed exercise or physical activity is termed delayed onset muscle soreness, or DOMS. The pain associated with DOMS often gets worse in the 48-72 hours following exercise before slowly reducing. The pain is thought to be caused by micro trauma to the muscle fibres. You're particularly likely to get DOMS if you've been walking or running up and down hills. Have you ever experienced DOMS?"

Participant responds:

Experimenter: Responds to the participant's answer and adds:

"While DOMS can be quite painful, the muscles adapt quickly. If you get DOMS once from some exercise you probably won't get DOMS the second time you do that exercise – or at least not nearly so bad. It just indicates that our muscles are

repairing after some unaccustomed exercise and are getting stronger. While most people know about how good regular exercise is for things like managing weight, improving lung and heart function, and even our mood, what we are starting to learn is that even a single bout of exercise can provide physical and psychological benefits. Have you come across much information about the benefits of just a single bout of exercise?"

Participant responds:

Experimenter: summarises key points raised by the participant and affirms these with the participant:

Participant responds:

Experimenter: reflects on any relevant points the participant has raised and then:

"Yes, some research has shown that even short bouts of exercise, as little as 5 minutes, can improve fitness, health and wellbeing. However, some people who decide to commence more strenuous exercise experience acute muscle soreness or DOMS and this often discourages them from further participation in exercise. This study that you have volunteered to take part in is about pain during a bout of normal

intensity exercise for 5 min and requires me to explain a bit more to you about pain during this type of exercise. I'll start by asking you a question. When you exercise, how do you determine if it is safe to continue if your pain increases or do you see the increase in pain as an indication to stop?"

Participant responds:

Experimenter: summarise any relevant information provided and then:

"It's normal to feel an increase in discomfort during exercise and this is not an indication that you are causing further damage to the muscle or that you are hurting yourself. It is safe to continue to exercise when the increases in pain you experience are tolerable and feel manageable. This discomfort should level out during exercise and reduce shortly after you finish. If you feel the muscles are

getting too tired or hurting too much during the exercise, then you should just drop the intensity slightly back to an easier level. You can apply this to the exercise bout you are about to undertake for this study. Before we continue, do you have any questions about pain and discomfort during exercise?"

Participant responds:

Experimenter: Answers any of the questions raised by the participant and then:

"I would now like to quickly talk about how levels of pain and exertion are typically measured during exercise. Do you know anything about this?"

Participant responds:

Experimenter: reflects on any relevant points the participant has raised and then:

"Because pain and exertion are both subjective sensations, they are normally assessed using self-report scales. For example, I might ask you to rate your pain during exercise on a 0 to 10 scale whereby 0 is no pain and 10 is the worst possible pain. I could use a similar scale to ask you about your level of exertion during exercise, and this information would be useful for me to know how hard you are finding the exercise. Have you used these types of scales before?"

Participant responds:

Experimenter: Answers any of the questions raised by the participant and then:

"The next thing I would like to discuss is something called exercise-induced hypoalgesia. Do you know anything about this?"

Participant responds:

Experimenter: Acknowledges any key points and then, with the aid of a 2, provides the following explanation:

"Exercise-induced hypoalgesia refers to a decrease in pain following exercise. A lot of studies show that this happens in both men and women and can last for

about 30 minutes following exercise. So, when we ask a person to rate their level of knee pain before exercise it is typical that their rating of pain after exercise has dropped. This can happen following resistance exercise, walking, cycling, or running exercise and it tends to happen more if we exercise longer or harder. We don't know exactly what causes exercise-induced hypoalgesia, but it seems to involve the release of substances within the body that reduce pain. Endorphins or natural opioids are the most obvious example that you might have heard of. Have you heard of endorphins?"

Participant responds:

Experimenter: Acknowledges any key points and then, with the aid of Image 2:

"These endorphins, along with other changes that occur with exercise, act to reduce the stress signals sent from the exercising muscles to the brain. The end result of this is that you experience less pain. This exercise analgesia might be why exercise is such an effective treatment for people with knee osteoarthritis. It's kind of a neat thing that the body becomes a little less sensitive to pain during exercise as it helps us to keep moving longer and to work harder. It's really cool that this effect lasts for a bit after we stop exercising, kind of like taking a painkiller. Do you have any questions about exercise-induced hypoalgesia?"

Patient responds:

[Experimenter presents and explains diagram of how exercise might act at various points in the pain pathway to reduce pain]

Experimenter: Responds to any questions then:

"Okay, well it's good that you are now a little more familiar with some of the causes of pain during exercise as well as how exercise can reduce pain and how this might be measured as you are going to be asked to rate these sensations when you are exercising later on. Is it ok if I summarise the key points we talked about before we commence exercise?"

Key points on a card:

- It is normal to experience some muscle pain and discomfort during and for a short time following exercise and pain during exercise doesn't mean that you are causing lasting damage or injury.

- It is also normal to experience longer lasting muscle pain, or DOMS, after intense or unaccustomed exercise, but the muscles gradually adapt to this and the pain subsides.

- During exercise at an intensity that causes some discomfort, tolerable increases in pain are normal and safe.

- Exercise-induced hypoalgesia is a reduction in pain that occurs after exercise and this can last for up to 30 min following exercise. It is common to experience exercise-induced hypoalgesia following resistance exercise like an isometric contraction, particularly when exercise is performed at higher intensities

"I just have a few final questions before we go on with the rest of the experiment."

[Experimenter presents the 5 questions below and asks the participant to indicate their level of agreement with each question. The experimenter then circles their response]

Experimenter: *"Thank you. Do you have any questions before we commence?"*

Participant responds:

Experimenter: *"Alright, well I hope that information was useful for you and that you have learned something about pain during exercise as well as how exercise can help to acutely reduce pain. Now I would just like to quickly explain what is going to*

happen for the rest of the experiment, after which I will hand over to one of my colleagues who will take you through the exercise bout and some pain assessments."

Following description of experimental procedures:

Experimenter: *"Thank you again for your time and for agreeing to participate in this*

study. I will leave you with my colleague and see you again when you're done."

Exercise Induced Hypoalgesia

<u>What is it?</u>

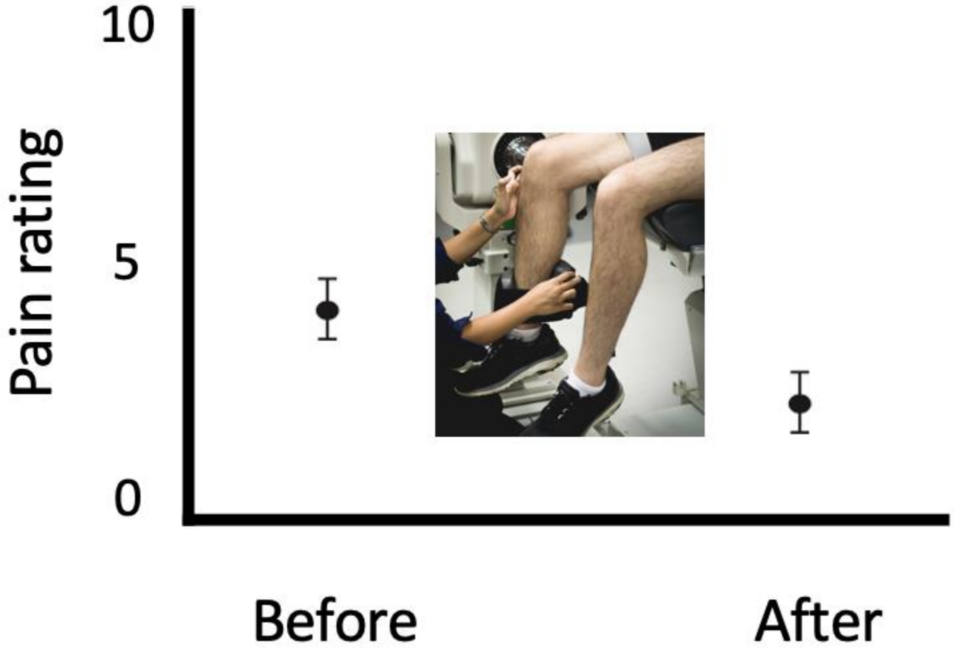

Why does it occur?

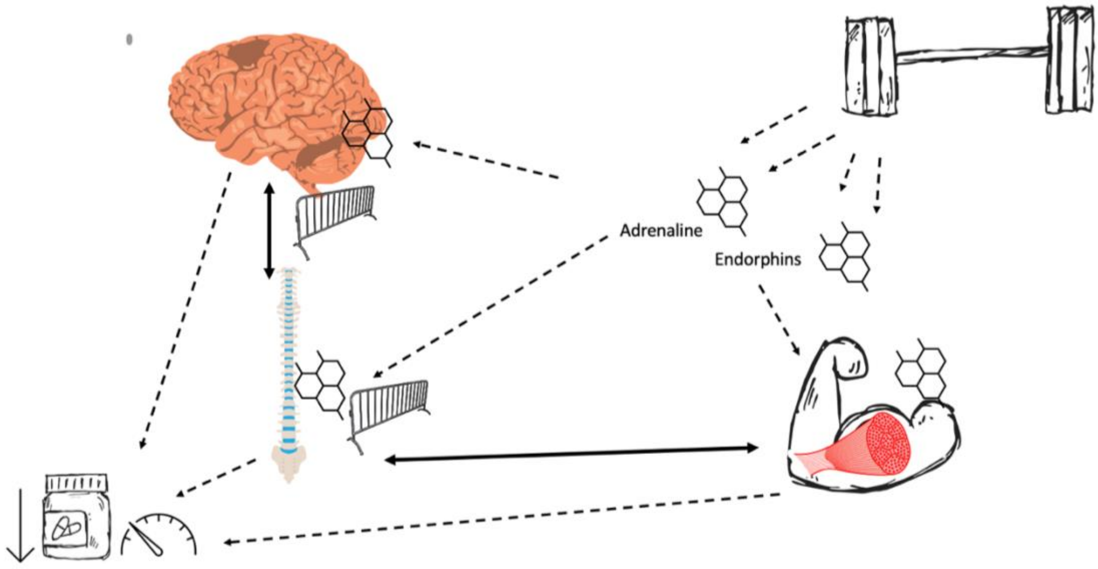

Supplement: Supplementary file 1 — Appendix S1: Active Control: Script for the education of participants with knee osteoarthritis, utilising the “Versus Arthritis: Osteoarthritis of the knee” handbook, excluding explicit information of exercise induced hypoalgesia. [file APL-29-e70587-s001.pdf]
